# The pattern of stressful life events prior to suicide among the older adults in rural China: a national case-control psychological autopsy study

**DOI:** 10.1186/s12877-020-01874-4

**Published:** 2020-11-16

**Authors:** Qiqing Mo, Zhenyu Ma, Guojun Wang, Cunxian Jia, Lu Niu, Liang Zhou

**Affiliations:** 1 School of Public Health, Guangxi Medical University, Nanning, Guangxi China; 2 Shenzhen Graduate School, Peking University, Shenzhen, Guangdong China; 3 School of Public Health, Shandong University, Jinan, Shandong China; 4 The Affiliated Brain Hospital of Guangzhou Medical University (Guangzhou Huiai Hospital), No. 36, Mingxin Road, Guangzhou, 510370 Guangdong China

**Keywords:** Chinese rural older adults, Stressful life event, Psychological autopsy, Suicide

## Abstract

**Background:**

There is a lack of evidence concerning the stressful life events experienced prior to suicide which may be associated with an increased suicide risk among Chinese rural older adults. The aim of this study was to identify the pattern of stressful life events prior to suicide among the older adults in China.

**Methods:**

Twelve counties were randomly selected using two-stage stratified cluster sampling method. Suicide cases aged 60 years and older (*n* = 242) were collected from those counties from June 2014 to September 2015. Matched living controls were selected 1:1 with suicide cases by age, gender, and location. Data were collected using face-to-face interviews by a psychological autopsy method. The Life Event Scale for the Elderly was used to measure the stressful life events prior to suicide/interviews.

**Results:**

Approximately 99.6% of suicide cases and 88.4% of controls experienced at least one stressful life event. The suicide group experienced more long-term stressful life events than recent stressful life events. The top three most frequent stressful life events for the suicide group were being diagnosed with chronic disease, hospitalization, and being diagnosed with terminal illness. More female suicide cases experienced the death of a spouse, while more males experienced hospitalization, diagnosis with terminal illness and family poverty. Experiencing at least one stressful life event, an unstable marital status, physical diseases and mental disorders were shown to increase the risk of suicide.

**Conclusions:**

Stressful life events were common for the rural older adults, especially long-term stressful life events. The experience of at least one stressful life event can increase suicide risk among this population. More attention should be paid to the rural older adults who experienced more long-term stressful life events and health related life events.

## Background

Suicide is a major public health and mental challenge for people worldwide. In 2006, the global age-standardized suicide rate was 10.6 per 100,000. The suicide rate is the highest among people aged 70 years and older for both men and women in almost every region in the world [1]. In China, the age-standardized suicide rate (per 100, 000) was 8.0 in 2016 [1]. In the age groups 60–69, 70–79, 80+ years, the suicide rates were 23.4, 44.0, and 61.3 [2], respectively, approximately three to eight times higher than the suicide rate in the general population. Furthermore, the suicide rates in rural areas are higher than those in urban areas in China [3]. Considering the pressure of the aging population, suicide prevention among the older adults in rural China is of great significance.

Family problems, work stress and physical diseases are the major stressful life events that most people experience [4]. These major stressful life events were reported to be high-risk factors for suicide through psychological strain [5–7]. An existing population-based study reported that stressful life events are positively associated to suicide [8] and that they may increase the probability of people killing themselves.

A number of empirical studies have identified stressful life events as factors that increase suicide risk [9]. Life events have been linked to suicide among younger and older individuals and among Western and Chinese youth [7]. The relationship between stressful life events and suicide suggests that stressful life events may be associated with suicide among Chinese rural older adults too, which has not been investigated yet [10].

The frequency of stressful life events is an important aspect related to the risk of suicide. One previous study reported that suicide risk increased with the frequency of these events [11]. People who experience more stressful life events are more likely to kill themselves. Routine risk assessments of vulnerable groups and an assessment of the variety of events which may indicate which individuals may be at immediate risk are necessary [12].

The relationship between stressful life events and suicidal behaviors among vulnerable people has been well studied, except among Chinese rural older adults. To the best of our knowledge, no study has ever focused on the stressful life events happening prior to suicide among Chinese rural older adults. The purpose of this study was therefor to summarize the pattern of stressful life events prior to suicide among older adults living in rural areas using psychological data.

## Methods

### Participants and procedures

A two-stage stratified cluster sampling method was used to select research sites. Three provinces were randomly selected from the GDP ranking of the top 10, 11–20, 21–31 provinces in mainland China these are Shandong, Hunan, and Guangxi. Counties were divided into three strata based on the average income within each province. Counties were randomly selected from each stratum, and a total of 12 counties were selected as research sites. A case-control psychological autopsy study was devised and implemented from June 2014 to September 2015 [13, 14]. A detailed description of the sampling methods, participants, and interview procedures was reported in our previous publication [15].

In each of the selected counties, suicide cases aged 60 years and older were collected consecutively from the death certification system. The controls were community members matched 1:1 with suicide case by age (± 3 years), gender, and location. For every participant, two informants were selected to obtain the data related to them. Generally, the first informant was a family member, and the second informant was a friend, a neighbor, or other relevant people. The survey was conducted through face-to-face interview with an average interview time of 90 min. For each pair of suicide and control, 4 interviews were conducted; a total of 968 interviews were completed for 242 pairs.

The investigators consisted of teachers and graduate students from Shandong University, Central South University and Guangxi Medical University. All investigators were trained intensively for 10 days in a standardized way on the instruments and the skills necessary for the interview.

The study was approved by the Institutional Review Board of the Shandong University, Central South University, and Guangxi Medical University. The aim and procedure of the research were explained to all participants. Written informed consent was obtained before interviews were conducted.

### Measurements

#### Demographic characteristics

Demographic variables included age (in years), gender (male/female), marital status (stable/unstable), family income (Yuan), presence of physical disease (yes/no), presence of mental disorder (yes/no), living alone (yes/no) and left behind by children (yes/no). In the study, people who were married and living with a spouse, or cohabitation with a partner were classified as “stable marital status”, while people with other marital status were classified as “unstable”.

#### Stressful life events for the older adults

Stressful life events were measured by the Life Events Scale for the Elderly (LESE), which was developed specifically for older Chinese adults [16]. LESE is a valid and reliable scale among the older adults in general, and for older adults who died by suicide in particular [16, 17]. A total of 46 life events were categorized into three separate categories: (1) Health/Hospital (16 items); (2) Family/Home (18 items); and (3) Friends/Relationships (12 items). Each life event was assessed by five questions: (1) The date it happened (never occurred, 1 month, 1 year, and more than 1 year ago); (2) Whether it was positive or negative for the participant; (3) The effect on participants’ mental health, measured by a five-point Likert scale from 1 = no impact, to 5 = very severe impact; (4) The duration of the event (3 months, 6 months, 1 year, and more than 1 year); and (5) The number of times it happened.

It should be noted that both positive and negative life events can cause a psychological stress response in participants. Therefore, stressful life events included positive as well as negative life events. In this study, stressful life events that happened within 1 month before suicide/interview for living control were converted to recent stressful life event. Stressful life events that happened within 1 year (excluding those within 1 month) or more than 1 year were converted to long-term stressful life events.

### Statistical analysis

The information provided by two informants was combined as proxy data for the suicides and controls. The demographic characteristics based on the information provided by the first informant was used. Answers associated with an increased risk of suicide were used when two informants reported differently for each item of LESE. The rationale for this practice is that a targeting behavior may exist if one of the two informants has observed it.

All participants were divided into two groups using the median number of stressful life events. The t-test and chi-squared test were used to compare the differences in demographic characteristics between the older adults who experienced fewer life events and more life events, for both among suicides and living controls. A chi-squared test was used to analyze the incidence of 46 stressful life events between suicides and controls. A chi-squared test was also used to compare the frequency of the top 10 stressful life events between males and females among the suicide cases. Logistic regression analysis was used to explore the relationship between stressful life events and suicide.

All analyses were performed using SPSS 24.0 for Windows (SPSS Inc., Chicago, IL). In this study, statistical significance was set as *P* less than 0.05.

## Results

### Demographic characteristics of suicides and controls by number of stressful life events

A total of 242 suicide cases and 242 living controls were included in this study. The number of stressful life events prior to suicide/interview ranged between 0 and 16 with a median value of 4 (Interquartile Range = 5) among all participants. As shown in Table 1, suicide cases and controls were divided into two groups according to the median number of stressful life events they experienced. There were no significant differences in most demographic characteristics between suicide cases experiencing more stressful life events and those experiencing fewer. Individuals who died by suicide who were diagnosed with mental disorders or left behind by children experienced more stressful life events than those without a mental disorder or who were not left behind by children. Living controls with unstable marital status, who lived alone, had physical diseases or were left behind by children experienced more stressful life events than controls with a stable marital status, who lived with others, did not have physical diseases, or had not been left behind by children.

**Table 1:** Demographic characteristics between suicides and controls by number of stressful life events (SLE)

Variables	Suicide cases			Controls		
	SLE ≤ 4	SLE > 4	*χ*^2^/t	SLE ≤ 4	SLE > 4	*χ*^2^/t
	n (%)	n (%)		n (%)	n (%)	
Age [mean (SD)]	73.6(8.6)	75.0(8.0)	1.268	73.0(8.4)	75.8(7.5)	2.731^*^
Sex						
Male	49(50.0)	86(59.7)	2.235	80(54.4)	55(57.9)	0.282
Female	49(50.0)	58(40.3)		67(45.6)	40(42.1)	
Marital status						
Stable	52(53.1)	70(48.6)	0.462	112(76.2)	58(61.1)	6.327^*^
Unstable	46(46.9)	74(51.4)		35(23.8)	37(38.9)	
Family annual income(yuan)						
0–3600	37(37.8)	51(35.4)	5.312	49(33.3)	25(26.3)	3.122
3601–10,000	28(28.6)	60(41.7)		53(36.1)	45(47.4)	
above 10,001	33(33.7)	33(22.9)		45(30.6)	25(26.3)	
Living alone						
Yes	23(23.5)	41(28.5)	0.75	14(9.5)	21(22.1)	7.384^*^
No	75(76.5)	103(71.5)		133(90.5)	74(77.9)	
Physical diseases						
Yes	78(79.6)	124(86.1)	1.796	86(58.5)	75(78.9)	10.831^*^
No	20(20.4)	20(13.9)		61(41.5)	20(21.1)	
Mental disorders						
Yes	41(41.8)	81(56.3)	4.846^*^	7(4.8)	5(5.3)	0.031
No	57(58.2)	63(43.8)		140(95.2)	90(94.7)	
Left behind by children						
Yes	10(10.2)	31(21.5)	5.314^*^	11(7.5)	14(14.7)	3.278^*^
No	88(89.8)	113(78.5)		136(92.5)	81(85.3)	

*: *Ρ* < 0.05; *SLE* Stressful life event; 4: the median number of stressful life events

### Stressful life events by time among suicide cases and living controls

The incidence of recent stressful life events and long-term stressful life events for suicide cases were 32.6 and 98.3% respectively, while the incidences for controls were 17.8 and 86.4%. As shown in Table 2, the incidence of recent stressful life events and long-term stressful life events for suicide cases were both significantly higher than for controls (*P* < 0.05).

**Table 2:** Stressful Life events by time among the suicide cases and living controls

Number of stressful life event	Suicide cases	Controls	*χ*^2^	*P*
	n (%)	n (%)		
Recent stressful life event				
0	163(67.4)	199(82.2)	14.743	0.002
1	48(19.8)	29(12.0)		
2	21(8.7)	10(4.1)		
≥ 3	10(4.1)	4(1.7)		
Long-term stressful life event				
0	4(1.7)	33(13.6)	44.118	< 0.001
1	18(7.4)	36(14.9)		
2	21(8.7)	36(14.9)		
≥ 3	199(82.2)	137(56.6)		

### Comparison of all items of stressful life events between suicide cases and living control

The LESE comprises 46 items for life events. As presented in Table 3, the incidences of most stressful life events among suicide cases was higher than among controls. The incidences of two categories of stressful life events, which were Health/Hospital and Family/Home were significantly higher among the suicide group than among the controls. Health/Hospital was the most reported for both suicide cases and living controls (97.5 and 83.9% respectively).

**Table 3:** Comparison of all items of stressful life events between the suicide cases and living controls

Stressful life events		Suicide cases	Controls	*χ*^2^	*Ρ*
		n (%)	n (%)		
Health/Hospital		236(97.5)	203(83.9)	26.681	< 0.001
1	Being diagnosed with chronic diseases	191(78.9)	141(58.3)	23.977	< 0.001
2	Being diagnosed with terminal illness	112(46.3)	43(17.8)	45.187	< 0.001
3	Life threatening illness to parents/spouse/children	48(19.8)	63(26.0)	2.63	0.105
4	Being injured from accident	21(8.7)	20(8.3)	0.027	0.87
5	Family members being injured from accident	27(11.2)	24(9.9)	0.197	0.657
6	Hospitalization	132(54.5)	84(34.7)	19.264	< 0.001
7	Family members hospitalized	44(18.2)	63(26.0)	4.331	0.037
8	Self-caring hard	71(29.3)	21(8.7)	33.551	< 0.001
9	Family members self-caring hard	20(8.3)	16(6.6)	0.48	0.488
10	Recovering from illness	12(5.0)	18(7.4)	1.279	0.258
11	Family members recovering from illness	8(3.3)	19(7.9)	4.746	0.029
12	Life threatening illness to relatives /close friends	5(2.1)	13(5.4)	3.693	0.055
13	Death of a spouse	77(31.8)	49(20.2)	8.412	0.004
14	Death of a child	29(12.0)	35(14.5)	0.648	0.421
15	Death of a son/daughter- in law	13(5.4)	11(4.5)	0.175	0.675
16	Death of a relative/close friend	14(5.8)	32(13.2)	7.783	0.005
Family/Home		172(71.1)	126(52.1)	18.477	< 0.001
17	Quarreling/Fighting with partner	32(13.2)	10(4.1)	12.619	< 0.001
18	Living apart from spouse	7(2.9)	4(1.7)	0.837	0.36
19	Legally divorced	1(0.4)	0(0.0)	–	1.000^#^
20	Being unfaithful	4(1.7)	0(.0)	–	0.123^#^
21	Spouse being unfaithful	2(0.8)	1(0.4)	–	0.624^#^
22	Being reconciled with spouse	0(0.0)	1(0.4)	–	1.000^#^
23	Children quarreling with their partner	23(9.5)	6(2.5)	10.601	0.001
24	Family poverty	67(27.7)	60(24.8)	0.523	0.47
25	Living alone	65(26.9)	32(13.2)	14.041	< 0.001
26	Crowded housing	9(3.7)	9(3.7)	0.000	1.000
27	Major loss in home/property	10(4.1)	15(6.2)	1.054	0.304
28	Children having left home	65(26.9)	69(28.5)	0.165	0.684
29	Disobedience of children	27(11.2)	10(4.1)	8.457	0.004
30	Daily life out of routine	29(12.0)	4(1.7)	20.325	< 0.001
31	Poor relationship with family members	42(17.4)	8(3.3)	25.784	< 0.001
32	Children unemployed	6(2.5)	11(4.5)	1.524	0.217
33	Major change in living conditions	3(1.2)	5(2.1)	–	0.724^#^
34	Major improvement in economic status	1(0.4)	3(1.2)	–	0.623^#^
Friend/Relationship		63(26.0)	47(19.4)	3.012	0.083
35	Tensions/Quarrying with neighbors	21(8.7)	18(7.4)	0.251	0.616
36	Separating/Breaking up with close friends	1(0.4)	2(0.8)	–	0.624^#^
37	No close friends/Loneliness	31(12.8)	12(5.0)	9.214	0.002
38	Retirement/Leaving the job	3(1.2)	6(2.5)	–	0.504^#^
39	Spouse retirement/Leaving the job	0(0.0)	4(1.7)	–	0.123^#^
40	Having legal trouble	3(1.2)	6(2.5)	–	0.504^#^
41	Family members having legal trouble	3(1.2)	7(2.9)	–	0.339^#^
42	Face-loss	11(4.5)	7(2.9)	0.493	0.483
43	Being misunderstood/misjudged	4(1.7)	8(3.3)	1.367	0.242
44	Being threatened	8(3.3)	4(1.7)	1.367	0.242
45	Family members being threatened	1(0.4)	1(0.4)	–	1.000^#^
46	Being deceived	1(0.4)	4(1.7)	–	0.217^#^
All life events		241(99.6)	214(88.4)	26.740	< 0.001

#:Fisher’s Exact Test

### Comparison of top 10 stressful life events by gender among suicide cases

Table 4 shows that the top three most reported stressful life events in the suicide group were being diagnosed with a chronic disease, hospitalization, and being diagnosed with a terminal illness. The same three most reported stressful life events were also observed in males. The top three stressful life events for female suicide cases were being diagnosed with a chronic disease, hospitalization, and the death of a spouse. More females experienced the death of a spouse than males. The frequency of hospitalization, being diagnosed with a terminal illness, and family poverty among males were significantly higher than in females.

**Table 4:** Comparison of top 10 stressful life events between females and males among the suicide cases

Stressful life events		Total	Rank	Female	Rank	Male	Rank	*χ*^2^
		n (%)		n (%)		n (%)		
1	Being diagnosed with chronic diseases	191(78.9)	1	87(81.3)	1	104(77.0)	1	0.655
6	Hospitalization	132(54.5)	2	48(44.9)	2	84(62.2)	2	7.257^*^
2	Being diagnosed with terminal illness	112(46.3)	3	40(37.4)	4	72(53.3)	3	6.108^*^
13	Death of a spouse	77(31.8)	4	46(43.0)	3	31(23.0)	8	11.036^*^
8	Self-caring hard	71(29.3)	5	26(24.3)	7	45(33.3)	5	2.350
24	Family poverty	67(27.7)	6	21(19.6)	9	46(34.1)	4	6.223^*^
25	Living alone	65(26.9)	7	29(27.1)	5	36(26.7)	6	0.006
28	Children having left home	65(26.9)	8	28(26.2)	6	37(26.4)	7	0.047
3	Life threatening illness to parents/spouse/children	48(19.8)	9	23(21.5)	8	25(18.5)	10	0.333
7	Family members hospitalized	44(18.2)	10	19(17.8)	10	25(18.5)	10	0.023

*: *Ρ* < 0.05

### Factors for suicide among the Chinese rural older adults

A total of 484 older adults were enrolled in the logistic regression model. After adjusting the variables of living alone and left behind by children, the model identified four factors that were all significant in increasing the risk of suicide among Chinese rural older adults, as shown in Table 5. Older adults with an unstable marital status, physical diseases, or mental disorders, or who have experienced at least one stressful life event were more likely to kill themselves.

**Table 5:** The analysis of influence factors for suicide among Chinese rural older adults

Variables	cOR (95% CI)	*P*	aOR (95% CI)	*P*
Marital status	2.322 (1.599–3.374)	< 0.001	2.527 (1.479–4.317)	0.001
Family annual income	1.129 (0.899–1.418)	0.296	–	–
Living alone	2.126(1.345–3.362)	0.001	1.258 (0.668–2.370)	0.478
Physical disease	2.541 (1.650–3.912)	< 0.001	1.874 (1.092–3.218)	0.023
Mental disorder	19.486 (10.350–36.687)	< 0.001	20.311 (10.391–39.700)	< 0.001
Left behind by children	1.771 (1.039–3.017)	0.036	0.924 (0.466–1.832)	0.820
Stressful life events	31.533 (4.254–233.728)	0.001	12.820 (1.578–104.175)	0.017

*cOR* crude odds ratio - Single factor analysis

*aOR* adjusted odds ratio - Multiple factors analysis

## Discussion

The major findings in the current study were: (1) Chinese rural older adults left behind by children experienced more stressful life events; (2) the incidence of long-term stressful life events were higher than that of recent stressful life events for the rural older adults; (3) the most common stressful life events for suicide cases were being diagnosed with a chronic disease, hospitalization, and being diagnosed with a terminal illness; (4) experiencing at least one stressful life event, having an unstable marital status, having physical diseases, and being diagnosed with mental disorders were the factors that increasing the probability of suicide among rural older adults in China.

The older adults who experienced at least one stressful life event were at higher risk to kill themselves. This finding also has been reported by previous studies [18]. Furthermore, for rural older adults, a great number of existing empirical studies support that stressful life events are associated with increased psychosocial distress and poor pain management [19, 20]. The experiences of new stressful situations can lead to suicidal behaviors [21]. The older adults also lived in worse conditions and this seemed to increase the risk of suicide. People at risk of suicide are not sensitive to a particular event that triggers the suicide, but to a whole set of living conditions, many of which contribute to suicidal behavior [22]. This study emphasizes the important role of stressful life events in suicide among Chinese rural older adults.

Long-term stressful life events were more common than recent stressful life events for both suicide cases and controls. Stress from life events accumulated and increased the risk of suicide. Accompanying increasing age, some stressful life events may always be present, such as physical illness, death of a spouse, and poverty. The incidence of at least one long-term stressful life event for older suicide cases was higher than that of younger suicide cases in rural China. The impact of long-term stressful life events on their suicide decision may be more profound. In our study, approximately 98.3% of suicide cases experienced a long-term stressful life event, and 32.6% of suicide cases experienced recent stressful life events. However, 82.4% of younger suicide cases experienced long-term stressful life events and 41.8% experienced recent stressful life events in an early psychological autopsy study in Chinese rural areas [23]. The difference between the incidences of long-term and recent stressful life events among younger and older rural individuals may indicate the impulsive personalities of young and old suicide cases. More attention needs to be paid to long-term stressful life events of rural older adults to reduce their stress.

The results suggested that the older adults left behind by children experience more stressful life events. Similar results were reported by a previous study. Being left behind by children parallels long-term separation and lack of social support, company, and care from family members, which may be profound in itself [19]. Nowadays, most rural young people leave their parents and hometown for a better life. The older adults left behind are more vulnerable to many stressful life events, such as self-care difficulties, family poverty, and children leaving home. Moreover, rural older adults living in rural area are more likely to experience various stressful life events due to a worse quality of life and a smaller social circle, as well as a poorer self-perceived health status [15, 24].

The most common stressful life events for elderly suicide in rural China were health related. The top three frequently stressful life events among suicide cases were being diagnosed with a chronic disease, hospitalization, and being diagnosed with terminal illness. When people become old, physical diseases cannot be avoided due to the natural aging process. Similar to previous literatures, the current study supported the presence of psychical illness as a factor that increases suicide risk [25]. Older adults with chronic diseases had more severe depressive symptoms, experienced stressful life events, had lower social support and a less positive coping method [26]. Taking care of the older adults with physical illnesses may be one method for reducing the risk of suicide associated with stressful life events.

There are also differences between males and females when it comes to stressful life events. Females were more likely to experience the death of a spouse due to their higher life expectancy, while males experienced more hospitalization, were diagnosed with terminal illness and experienced family poverty. Family poverty, work problems, and family discord were more commonly reported among male suicide cases in Western society [7]. Unstable marital status was a risk factor of suicide. A spouse is always the most important and available caregiver during old age. Therefore, a stable marital status has a positive impact on the physical and mental health of older adults, both in terms of life care and emotional well-being [27]. The older adults without a spouse tend to lose spiritual comfort and gain less support than they would from a spouse [28].

There were some limitations to this psychological autopsy study. Firstly, recall bias is an inevitable limitation in such a retrospective investigation, in which interviewees report previous life events. Secondly, the proxy data were used to assess the stressful life events which happened prior to the suicide/interview. The accuracy of information should be further improved. Thirdly, the long-term impact of stressful life events has not been assessed. Further studies should focus on the long-term effect of stressful life events on elderly suicide.

## Conclusions

This study demonstrates the characteristics of stressful life events among suicides and the relationship between stressful life events and suicide. Stressful life events are factors that increase the risk of suicide. Long-term stressful life events were common among rural older adults. More attention should be paid to the rural older adults who experienced more long-term stressful life events and experienced health-related stressful life events to reduce the risk of suicide.

## Data Availability

The datasets used during the current study are available from the corresponding author on reasonable request.

## Abbreviations

LESE: Life event scale for the elderly
LE: Life event
CDC: Center for disease control and prevention
QR: Quantile range
cOR: Crude odds ratio
aOR: Adjusted odds ratio
